# Six Month *In Situ* High-Resolution Carbonate Chemistry and Temperature Study on a Coral Reef Flat Reveals Asynchronous pH and Temperature Anomalies

**DOI:** 10.1371/journal.pone.0127648

**Published:** 2015-06-03

**Authors:** David I. Kline, Lida Teneva, Claudine Hauri, Kenneth Schneider, Thomas Miard, Aaron Chai, Malcolm Marker, Rob Dunbar, Ken Caldeira, Boaz Lazar, Tanya Rivlin, Brian Gregory Mitchell, Sophie Dove, Ove Hoegh-Guldberg

**Affiliations:** 1 Scripps Institution of Oceanography, Integrative Oceanography Division, University of California San Diego, San Diego, California, United States of America; 2 Global Change Institute, The University of Queensland, Brisbane, Australia; 3 Coral Reef Ecosystems Laboratory, School of Biological Sciences, The University of Queensland, Brisbane, Australia; 4 The ARC Centre of Excellence for Coral Reef Studies, The University of Queensland, Brisbane, Australia; 5 Stanford University, Environmental Earth System Science, Stanford, CA, United States of America; 6 Conservation International, Betty and Gordon Moore Center for Science and Oceans, Honolulu, HI, 96825, United States of America; 7 Carnegie Institution, Department of Global Ecology, Stanford, CA, United States of America; 8 International Pacific Research Centre, University of Hawaii, Honolulu, HI, United States of America; 9 Institute of Marine Science, School of Fisheries and Ocean Sciences, University of Alaska Fairbanks, Fairbanks, AK, United States of America; 10 Institut Océanographique Paul Ricard, Ile des Embiez- Le Brusc, 83140, Six-Fours-Les-Plages, France; 11 The University of Queensland, Faculty of Engineering, Architecture and Information Technology, Brisbane, Australia; 12 The Interuniversity Institute for Marine Sciences, The H. Steinitz Marine Biology Laboratory, The Hebrew University of Jerusalem, Eilat, Israel; Leibniz Center for Tropical Marine Ecology, GERMANY

## Abstract

Understanding the temporal dynamics of present thermal and pH exposure on coral reefs is crucial for elucidating reef response to future global change. Diel ranges in temperature and carbonate chemistry parameters coupled with seasonal changes in the mean conditions define periods during the year when a reef habitat is exposed to anomalous thermal and/or pH exposure. Anomalous conditions are defined as values that exceed an empirically estimated threshold for each variable. We present a 200-day time series from June through December 2010 of carbonate chemistry and environmental parameters measured on the Heron Island reef flat. These data reveal that aragonite saturation state, pH, and pCO_2_ were primarily modulated by biologically-driven changes in dissolved organic carbon (DIC) and total alkalinity (TA), rather than salinity and temperature. The largest diel temperature ranges occurred in austral spring, in October (1.5 – 6.6°C) and lowest diel ranges (0.9 – 3.2°C) were observed in July, at the peak of winter. We observed large diel total pH variability, with a maximum range of 7.7 – 8.5 total pH units, with minimum diel average pH values occurring during spring and maximum during fall. As with many other reefs, the nighttime pH minima on the reef flat were far lower than pH values predicted for the open ocean by 2100. DIC and TA both increased from June (end of Fall) to December (end of Spring). Using this high-resolution dataset, we developed exposure metrics of pH and temperature individually for intensity, duration, and severity of low pH and high temperature events, as well as a combined metric. Periods of anomalous temperature and pH exposure were asynchronous on the Heron Island reef flat, which underlines the importance of understanding the dynamics of co-occurrence of multiple stressors on coastal ecosystems.

## Introduction

By 2100, tropical surface seawater temperatures are projected to rise by 2–3°C [1] and open ocean pH levels are projected to decline by 0.3–0.4 units [2]. Ocean warming can lead to thermal physiological stress in corals known as coral bleaching [3]. Increasing CO_2_ content in surface seawater leads to ocean acidification (OA) which can reduce skeleton- and shell-building ability in many marine organisms [4–7] and possibly even hamper reef ecosystem calcification for many reefs globally [8, 9]. The relevance of carbonate chemistry variability (pH, dissolved inorganic carbon [DIC], and total alkalinity [TA]) to coral physiology, reef ecology and susceptibility to OA is increasingly appreciated [10–13]. Current models predict a 60% decline in reef coral coverage within the next few decades due to warming, and a 10–50% drop in coral calcification and even a shift towards net reef dissolution by the end of this century due to OA [3, 14–17].

Compared to environmental variability in the open ocean, coral reefs are dynamic coastal ecosystems, with large diel and seasonal temperature and pH variability. Daily temperature swings are largely driven by cloud cover, currents, turbidity, tides and solar heating [18], and can range from 4.0 to 8.0°C (e.g., [11, 19]). Several studies suggest that the mean monthly and seasonal temperatures, as well as diel temperature changes or the short term thermal history, are important for determining coral bleaching temperature stress thresholds [11, 20]. It has also been suggested that daily exposure of corals to potentially stressful thermal conditions that persist long enough to induce acclimation but not to cause mortality may increase corals’ bleaching resistance [19]. Diel pH variability can be as large as 0.5–0.6 pH units, greater than the pH decline predicted by 2100 for the open ocean [21–23]. Such large diel ranges are driven by reef metabolism processes of photosynthesis/respiration and calcification/dissolution as well as physical controls on the local seawater residence time at the site [24, 25]. Seasonal pH ranges can also be substantial, up to 0.7–0.8 pH units, especially on isolated reef flats with long seawater residence time [22]. Seasons can substantively influence other environmental variables on reefs, such as temperature, light, and precipitation, while longer term inter-annual variation can be due to processes such as El Niño/La Niña events [26]. Furthermore, carbonate chemistry variability on reefs may have large implications in a high CO_2_ future due to predicted amplification of the variability [12].

A better grasp of potential variation in OA impacts across different reef zones and communities requires detailed characterization of their natural diel and seasonal range in carbonate chemistry (pH, pCO_2_, DIC, TA, and aragonite saturation state [Ω_AR_]). We use an accepted model of vulnerability in this study, wherein we consider the relative vulnerability of reefs to be a function of exposure to a hazard or risk and of sensitivity to such hazards or risks [27, 28]. A significant knowledge gap in our understanding of OA impacts on coral reefs lies in understanding the exposure of reefs to natural variability in OA-relevant parameters on different time-scales. This study aims to provide a high-resolution dataset, which delineates different patterns of natural variability in environmental parameters over 6 months on a coral reef. An upcoming publication will depict a study that tests both exposure and sensitivity to exposure in an OA experiment [29].

Most previous studies of carbonate chemistry dynamics on shallow coral reef environments have monitored the environment either long-term (months to years) with *in situ* pH sensors but often with few discrete measurements, or short-term (days) yet at high-resolution (>3–4 measurements per day) and sometimes repeated in different seasons (e.g., [22, 30, 31–33]). Several studies are attempting to determine the complex interaction between environmental factors (light, wave energy, temperature, ecological community composition, etc.), carbonate chemistry and reef function [25, 34] but often field and logistical challenges constrain such studies to short-term (several days) data collections of anomalously high-frequency carbonate system measurements (e.g. [30]).

This study presents one of the most high-resolution environmental data suites for a coral reef environment in a 6-month period, including 10-s water depth data, 10-s water velocity data, 10-s temperature data, 10-s salinity data, 10-s PAR, 10-s pH, and daily to hourly DIC and total alkalinity. Phosphate and nitrate data were also collected for 150 samples at nearly daily resolution during the 6 months of the study. This large dataset library for Heron Island provides valuable detail of environmental variability on various time-scales. This paper contributes: 1) documentation, in as high detail as logistically feasible, of the environmental variability on Heron reef flat with a specific focus on carbon system parameters and temperature; 2) an assessment of relative contributions to the natural variation in pH, aragonite saturation state, and pCO_2_ from thermodynamic and biological processes; and 3) development of a set of empirically derived pH and temperature metrics for a better understanding of reef environmental exposure over time. Periods of anomalous temperature and pH exposure were asynchronous on the Heron Island reef flat, which may have implications for overall reef habitat sensitivity and biological responses to future global ocean changes (see Methods section on how we have defined ‘anomalous’ within this dataset for each variable). This study suggests that high-resolution data sets in near shore environments are critical for understanding the dynamics of co-occurrence of multiple stressors on coastal ecosystems.

## Materials and Methods

### Ethics Statement

Permits from the Department of Environment and Resource Management (#CSCE00874010) and the Great Barrier Reef Marine Park Authority (#G09/29996.1) were provided to conduct this research.

### Study Site

**Fig 1:**
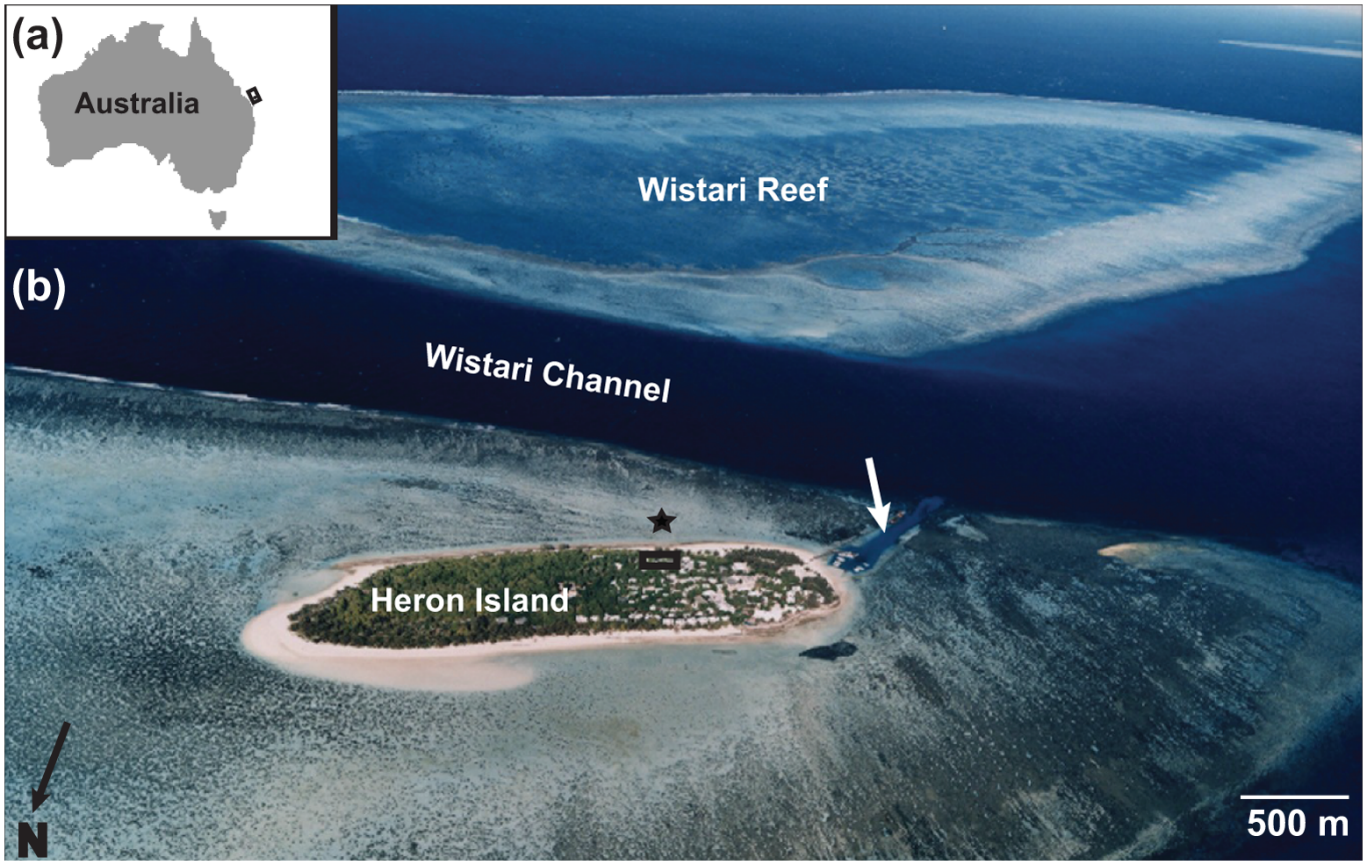
Location of the Heron Island reef flat research site. (a) Map of Australia with a black rectangle representing the Capricorn Bunker group of reefs at the southernmost end of the Great Barrier Reef with a circle for Heron Island. (b) Aerial photograph of Heron Island with the reef flat study site shown as a star, the Heron Island Research Station aquarium system as a black rectangle and the channel cut in the reef flat as a white arrow.

This study was conducted on the Heron Island reef flat (23° 27’ S, 151° 55’ E), a coral cay that is part of the Capricorn-Bunker Group of reefs at the southern end of the Great Barrier Reef (GBR, Fig 1). Corals of the *Acropora* and *Porites* genera cover >20% of the Heron Island reef flat [35]. The Heron Island reef flat has semi-diurnal tidal cycles, with a spring tidal range of 2.3 m, a neap tidal range of 1.1 m [36] and depths between 0.3–1 m in the shallowest reef flat areas at low tides when the reef flat is isolated by the emergent reef rim [37]. The Heron Island algal reef rim on the southwest side of the island was dredged to allow passage of boats [38] (Fig 1). This modification has altered the hydrodynamics of the reef flat waters resulting in continual flushing of reef waters through the dredged channel even at low tide. December through February, or austral summer, has the highest rainfall, whereas austral winter (June to September) is the driest season [36]. According to temperature data from the Heron Island weather station (ID 39122, Australian Bureau of Meteorology) for the 1962–2006 period, the air temperature has been warmest in January, at 29.7 ± 0.9°C (+/- STD), and coldest in July at 21.5 ± 0.8°C. Sea-surface temperatures available from the Australian Institute of Marine Sciences for 2008–2013 and derived from sensors across the Heron lagoon show similar trends to air temperature: January has the annual maximum of 29.4 ± 0.7°C and July has the minimum temperatures with an average of 23.3 ± 0.75°C [39]. Rainfall and wind data were also obtained from the Australian Bureau of Meteorology for the duration of the study [39].

### Instrumentation Array

The instrumentation array included a Monterey Bay Area Research Institute (MBARI) modified Sea-Bird 18 digital pH sensor sampling every second (1-second resolution, manufacturer’s reported precision of ± 0.001 pH units; field precision of approximately ± 0.01 pH units, Nido Instruments, USA), a Vector acoustic velocimeter (2-minute resolution; Nortek, Norway), and a Conductivity, Temperature, Depth (CTD) instrument (10-second resolution, SBE-16plusV2, Sea-Bird Electronics, USA) with a Satlantic Photosynthetically Active Radiation (PAR) sensor (Sea-Bird Scientific, USA). The instruments were attached to cinder blocks with PVC clips on the Heron Island reef flat (23°27’S, 151°55’E) in front of the research station approximately 50 m from shore at a submerged location, which ranged in tidal depth from 0.3 m to 2.5 m (Fig 1). The instrument array was connected to a waterproof CompacRIO computer pod (National Instruments, USA) and a 12V power supply (details of the instrumentation array can be found in [40, 41]). The pH sensors were swapped out with freshly calibrated sensors every 48–72 hours and cleaned daily. The pH sensors were calibrated to the seawater scale using a Denver pH meter (UB-10, Denver Instruments, NY, USA) with NBS standards and then used to prepare pH 6.00 and 8.20 filtered seawater standards. The mV readings of the pH sensors in the freshly prepared seawater standards were then used for sensor calibration to the seawater pH scale using a spreadsheet developed by MBARI. A powerful tropical storm in November 2010 disabled many of the field instruments and limited access to the site, resulting in a data gap in November.

### Discrete Measurements

Discrete samples for DIC, TA, and pH were collected at variable frequencies throughout the study. In June and December, availability of personnel allowed for the capture of the full diel cycle in DIC and TA through 3–4 hour interval sampling on the reef flat. During the winter and early spring months, we were constrained to sampling once or twice a day, typically at noon and at the daytime low tide, due to lack of personnel. DIC samples were measured with a LI-COR 7000 H_2_O:CO_2_ analyzer (LI-COR, Lincoln) coupled to an automated DIC sample introduction unit built by Stanford University’s Stable Isotope Laboratory. DIC precision ranged between ± 1–2 μmol kg^-1^, and the accuracy was determined with certified reference materials (CRMs) from A.G. Dickson (Scripps Institution of Oceanography, Oceanic Carbon Dioxide Quality Control). 226 DIC samples and 216 TA discrete samples were collected over the course of the study. TA was measured via potentiometric titrations in accordance with Gran titration procedures [42], using a T50 titrator with a small-volume DGi101-SC pH sensor (Mettler Toledo, Switzerland). All TA samples were corrected based on the offset between the measured and certified value of the CRM; TA precision was ± 3 μmol kg^-1^. DIC, TA, temperature and salinity were used to compute pCO_2_ and Ω_AR_, using CO2SYS [43]. K1 and K2 dissociation constants used were from Mehrbach et al. (1973) [44], as refitted by Dickson and Millero (1987) [45], and the constant for sulfate, KSO_4_, was from [46]. Monte Carlo simulations (5000 runs) provide error sensitivities of ± 3.5 ppm for pCO_2_, an error of ± 0.03 units for Ω_AR_, and ± 0.01 units for pH. We tested the sensitivities of outputs to input pairs and this analysis demonstrated that the derived values of TA are most sensitive to salinity changes. DIC and TA values were normalized to the 6-month average salinity of 35.2 psu and presented as nDIC and nTA. Non-normalized DIC and TA values were used with CO2SYS to compute aragonite saturation state and pCO_2_. Otherwise, we display salinity-normalized values for the 6-month period in order to remove the effects of rainfall and evaporation on the DIC and TA values and observe the effects of the benthos on the water column chemistry.

### Thermodynamic vs. Biological Controls on pH and Ω_AR_

Variability in pH and Ω_AR_ is driven by temperature and salinity as well as the relative concentrations of DIC and TA. Separately from the thermodynamic controls of temperature and salinity, the biological processes of net ecosystem calcification (NEC) and net ecosystem production (NEP) affect the TA/DIC ratio, which in turn affects the seawater pH and Ω_AR_ at the site [22, 47]. To understand the relative contribution of these different drivers on pH and Ω_AR_ variability, we performed a simple decomposition, previously described in [12, 48]:

$$\Delta pH_{\left(TOTAL\right)}=\frac{\partial pH}{\partial T}dT+\frac{\partial pH}{\partial S}dS+\frac{\partial pH}{\partial DIC}dDIC+\frac{\partial pH}{\partial TA}dTA$$ (1)

$$\Omega_{AR}=\frac{\partial \Omega_{AR}}{\partial T}dT+\frac{\partial \Omega_{AR}}{\partial S}dS+\frac{\partial \Omega_{AR}}{\partial DIC}dDIC+\frac{\partial \Omega_{AR}}{\partial TA}dTA$$ (2)

In each of these equations, the partial derivative accounting for the contribution of temperature, salinity, DIC, or TA was calculated by holding the other three components constant. The temperature, salinity, DIC, and TA values used in this were not hypothetical end-member values, but the data collected from Heron Island reef flat during December 2010 when there was a relatively high sampling resolution of 3–4 hours. This approach produces a time series of pH, Ω_AR_, and pCO_2_ along with relative contributions from temperature, salinity, DIC, and TA for the site.

### Environmental Exposure Metrics

The environmental exposure metrics of intensity, duration, and severity of events were computed for the pH and temperature data following Hauri *et al*. (2013) [49]. We use pH rather than Ω_AR_ values for the development of a carbonate system metric because the pH data was sampled at much higher resolution (10 s), whereas the Ω_AR_ values have been derived from relatively fewer discrete samples of DIC and TA over the duration of our study. While Ω_AR_ provides much useful, biologically relevant information, in this case, the high-resolution pH dataset is more suitable for intensity and severity analyses on different time-scales. We also developed a new combined exposure metric for intensity, duration, and severity that incorporated both pH and thermal exposure. Anomalous pH and temperature events were recorded as intensity events, which we determined by observing when, to what extent, and for how long values exceeded a predetermined threshold (Table 1). We used a pH threshold of 8.1, based on the 2010 mean global open ocean pH [31], and a temperature threshold based on the mean monthly maximum temperatures measured by the Australian Institute of Marine Science from 2008–2013 with sensors distributed across the Heron Island lagoon (S1 Table). The duration of the thus-defined anomalous event was estimated as the amount of time passed until 15-minute mean values of pH or temperature returned to the threshold levels after exceedence. The severity of each event (either a low-pH or high-temperature event) was calculated as the product of intensity and duration (total pH unit•hr, °C•hr, respectively). Therefore, each month had a number of low-pH as well as a number of high-temperature events.

**Table 1:** Environmental exposure metrics calculations.

Type of metric	Low pH exposure	High temperature exposure
Intensity of singular event (I)	*I*_*pH*_ = *TR*_*pH*_−*pH*_15−*min*_	*I*_*T*_ = *TR*_*T*_−*T*_15−*min*_
Duration of singular event (D)	Length of time until the pH returned to a value equal to the threshold	Length of time until the temperature returned to a value equal to the threshold
Severity of singular event (S)	*S*_*pH*_ = *I*_*pH*_×*D*_*pH*_	*S*_*T*_ = *I*_*T*_×*D*_*T*_
Mean monthly scaled intensity	$I_{pH-MEAN}\;=\;\frac{\sum_{i}^{N_{pH}}\left(I_{pH-i}∕I_{pH-MAX}\right)}{N_{pH}}$	$I_{T-MEAN}\;=\;\frac{\sum_{i}^{N_{T}}\left(I_{T-i}∕I_{T-MAX}\right)}{N_{T}}$
Mean monthly scaled duration	$D_{pH-MEAN}\;=\;\frac{\sum_{i}^{N_{pH}}\left(D_{pH-i}∕D_{pH-MAX}\right)}{N_{pH}}$	$D_{T-MEAN}\;=\;\frac{\sum_{i}^{N_{T}}\left(D_{T-i}∕D_{T-MAX}\right)}{N_{T}}$
Mean monthly scaled severity	$S_{pH-MEAN}\;=\;\frac{\sum_{i}^{N_{pH}}\left(S_{pH-i}∕S_{pH-MAX}\right)}{N_{pH}}$	$S_{T-MEAN}\;=\;\frac{\sum_{i}^{N_{T}}\left(S_{T-i}∕S_{T-MAX}\right)}{N_{T}}$
Combined monthly mean intensity	*C*_*I−MEAN*_ = *I*_*T−MEAN*_+*I*_*pH−MEAN*_	
Combined monthly mean duration	*C*_*D−MEAN*_ = *D*_*T−MEAN*_+*D*_*pH−MEAN*_	
Combined monthly mean severity	*C*_*S−MEAN*_ = *S*_*T−MEAN*_+*S*_*pH−MEAN*_	

Fifteen-minute temporal resolution data were used for the pH and temperature exposure metrics. The pH threshold was chosen to be 8.1 as this is the present average pH of the open ocean [72]. The temperature thresholds (TR_T_) were different for each month (June-December) and were equivalent to a Mean Monthly Maximum (MMM) time series we computed for the site based on field data for 2008–2013 (Australian Institute for Marine Science, http://data.aims.gov.au/aimsrtds/station.xhtml?station=130) (S1 Table). Any maximum values here represent maxima for the whole dataset rather than the maximum within a given month (e.g., I_pH-MAX_ is the maximum intensity across the 6 months of data).

A combined exposure metric, containing information on both pH and temperature exposure, required a rescaling of the intensity, duration, and severity values for each variable. For example, within a given month, pH intensity, duration, and severity values were separately divided by the maximum intensity, duration, or severity, respectively, to normalize values for every month on a 0–1 scale. The same scaling was applied for the temperature variable. Then, the scaled intensity, duration, and severity were each averaged per month, for both pH and temperature (see Table 1 for equations). The composite metrics for intensity (total pH unit•°C), duration (hr), and severity (total pH unit•°C•hr) for a given month were computed by adding the mean monthly scaled pH exposure intensity to the mean monthly scaled thermal exposure intensity.

### Statistical Analysis

All statistical analyses were done on a MatLab platform. Pearson’s correlation was used to test correlations between datasets. The Kruskal-Wallis test was used to test for significant difference across seasons for the same environmental variable. Kruskal-Wallis is the non-parametric version of one-way analysis of variance and does not assume normal distribution of datasets; therefore, this test is better suited for the task at hand.

## Results

### Variability in Environmental Parameters

#### Temperature

**Fig 2:**
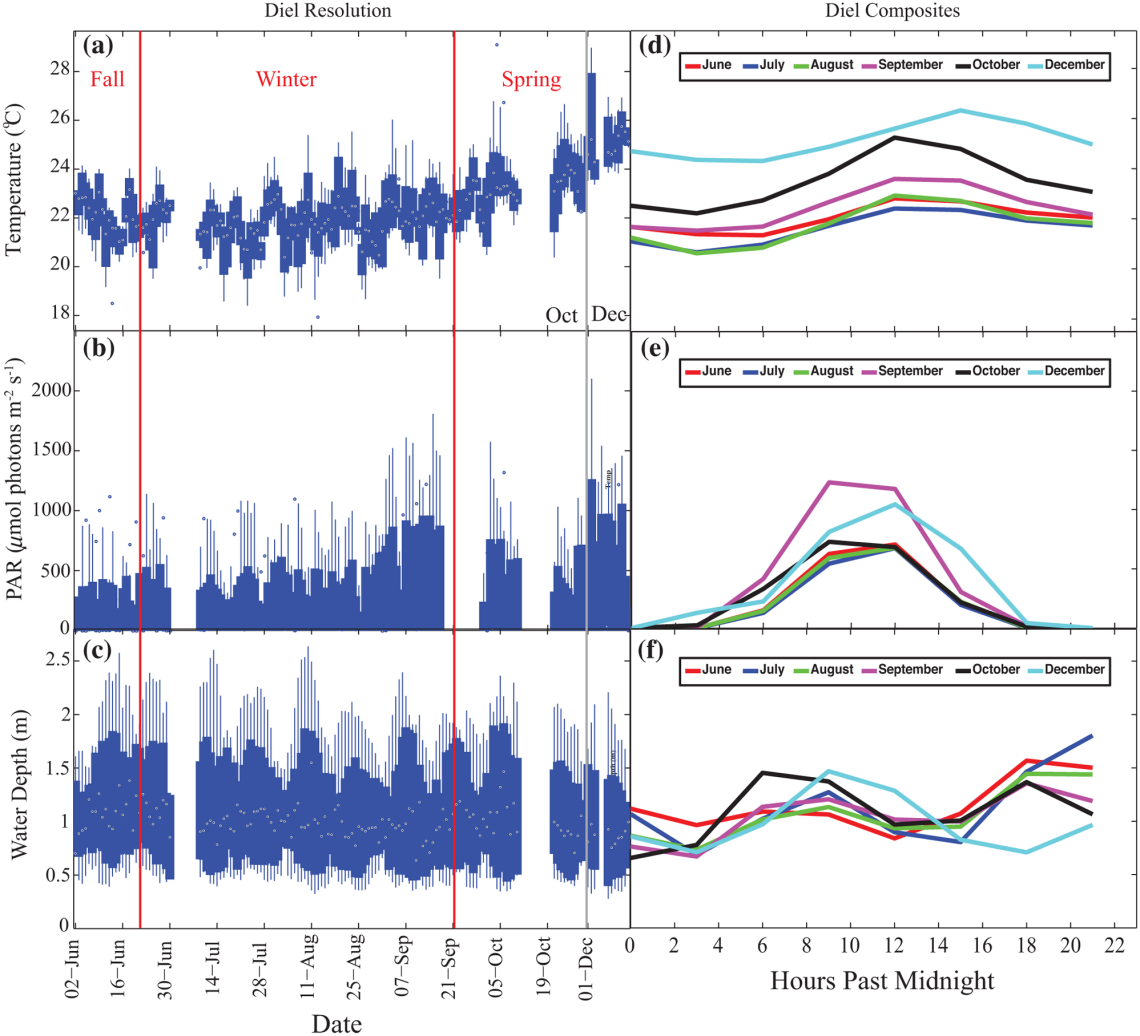
High resolution plots of temperature, light and water depth. (a) Box plots of temperature, (b) light and (c) water depth data with hourly resolution. The edges of the box are the mid-range (25–75^th^ percentile), the lines extend to the most anomalous data points not considered outliers, and outliers are plotted individually. (d) Diel composites shown for each month for temperature, (e) light and (f) water depth.

As expected, diel average temperatures had winter minima and increased during the spring (Fig 2A). The largest diel temperature ranges were in the spring, in October (1.5–6.6°C), and lowest diel averages were observed in July (0.9–3.2°C; Fig 2A). Diel composites of temperature variability for an average 24-hr period for each month reveal consistent thermal maxima around noon to shortly afternoon, and minima around 02:00, as well as an increase in the mean temperature with progression through winter and spring (Fig 2D). In this study, the highest diel temperature maxima of 26.3 ± 1.2°C were observed in December, towards the end of austral spring. The mean monthly seawater temperatures ranged from 22.0 ± 0.7°C in June to 25.1 ± 0.6°C in December 2010 (Tables 2 and 3). Temperature varied across seasons in a statistically significant way, as evidenced by Kruskal-Wallis tests (Table 4).

**Table 2:** Monthly environmental data summary statistics including the average diel means, average diel minimum, average diel maximum and diel range for each parameter (± SD).

		June	July	August	Sept	Oct	Dec
**Temp**.	Mean	22 ± 0.7	21.6 ± 0.7	21.8 ± 0.7	22.4 ± 0.6	23.5 ± 0.7	25.1 ± 0.6
	Min	20.9 ± 1	20.4 ± 1	20.3 ± 1	21.2 ± 0.8	22.2 ± 0.8	24.1 ± 0.6
	Max	23 ± 0.8	22.6 ± 0.9	23.2 ± 1	23.8 ± 0.9	25.4 ± 1.4	26.3 ± 1.2
	Range	0.02–3.6	0.9–3.2	1.1–5.1	0.7–4.8	1.5–6.6	0.4–4.9
**PAR**	Mean	167 ± 77	193 ± 54	205 ± 69	361 ± 96	256 ± 96	342 ± 187
	Max	1139 ± 327	1082 ± 231	1097 ± 257	1807 ± 294	1575 ± 305	2101 ± 579
**pH**	Mean	8.24 ± 0.04	8.1 ± 0.06	8.11 ± 0.08	8.04 ± 0.06	8.1 ± 0.04	8.05 ± 0.07
	Min	8.06 ± 0.08	7.8 ± 0.3	7.89 ± 0.13	7.78 ± 0.09	7.83 ± 0.08	7.78 ± 0.12
	Max	8.42 ± 0.1	8.31 ± 0.09	8.32 ± 0.08	8.27 ± 0.15	8.31 ± 0.08	8.3 ± 0.13
	Range	0.18–0.61	0.25–1.6	0.22–0.73	0.05–0.83	0.23–0.56	0.25–0.89
**nDIC**	Mean	1925 ± 45	1932 ± 83	1840 ± 109	1916 ± 60	1904 ± 94	1975 ± 89
	Min	1701 ± 76	1779	1603	1813	1752	1677 ± 107
	Max	2073 ± 39	2170	1990	2032	2072	2199 ± 120
	Range	372	391	387	219	320	522
**nTA**	Mean	2252 ± 22	2263 ± 22	2236 ± 33	2245 ± 37	2233 ± 33	2303 ± 30
	Min	2147 ± 35	2218	2156	2118	2172	2254 ± 26
	Max	2300 ± 18	2309	2283	2302	2268	2387 ± 49
	Range	153	111	127	184	96	133
**pCO**_**2**_	Mean	345 ± 53	349 ± 145	228 ± 74	326 ± 84	361 ± 142	362 ± 117
	Min	148 ± 70	203	100	200	193	139 ± 99
	Max	616 ± 70	563	371	491	659	882 ± 222
	Range	468	360	271	291	466	743
**Ω**_**AR**_	Mean	3.6 ± 0.3	3.6 ± 0.8	4.4 ± 0.8	3.6 ± 0.5	3.6 ± 0.7	3.8 ± 0.7
	Min	2.4 ± 0.3	2.5	3.2	2.5	2.3	2 ± 1
	Max	5.3 ± 0.5	4.7	5.9	4.5	4.6	5.6 ± 0.7
	Range	2.9	2.2	2.7	2	2.3	2.6

For temperature and pH, the range values for each month represent a span of the minimum diel and maximum diel range observed that month. For nDIC, nTA, pCO_2_ and Ω_AR_ the range given for the month is the difference between the observed daily maximum and minimum, except for June and December when the range is the difference between the averaged daily maximum and minimum. The standard deviations, where given, represent standard deviation around the diel mean, minimum and maximum for variables where the authors are confident that high-resolution diel data allows for the capture of the full diel cycle. Standard deviations are not given for months where nDIC, nTA, pCO_2_, Ω_AR_ are not resolved for the full diel cycle.

**Table 3:** Seasonal environmental statistics, with absolute minima and maxima observed.

Parameter		Fall	Winter	Spring
**Temperature**	**n**	*113*	*670*	*388*
	Mean	22.0 ± 1.1	21.9 ± 1.1	23.4 ± 1.5
	Min	18.5	17.9	20.4
	Max	24.1	26	29.1
	Range	5.6	8.1	8.7
**PAR**	**n**	*113*	*638*	*237*
	Mean	169 ± 273	232 ± 356	295 ± 414
	Max	1116.4	1806.7	2101
**pH**	**n**	*1429*	*6298*	*2525*
	Mean	8.24 ± 0.1	8.12 ± 0.14	8.04 ± 0.14
	Min	7.85	7.57	7.66
	Max	8.58	8.61	8.45
	Range	0.73	1.04	0.79
**nDIC**	**n**	*48*	*61*	*67*
	Mean	1913 ± 94	1931 ± 92	1952 ± 118
	Min	1701	1603	1677
	Max	2071	2073	2199
	Range	370	470	522
**nTA**	**n**	*48*	*72*	*50*
	Mean	2242 ± 39	2259 ± 31	2273 ± 56
	Min	2148	2156	2118
	Max	2298	2309	2387
	Range	150	153	269
**pCO**_**2**_	**n**	*55*	*66*	*48*
	Mean	344 ± 96	326 ± 126	374 ± 163
	Min	148	100	139
	Max	528	867	882
	Range	380	767	743
**Ω**_**AR**_	**n**	*56*	*66*	*48*
	Mean	3.6 ± 0.6	3.7 ± 0.7	3.6 ± 0.8
	Min	2.4	1.7	2
	Max	5.3	5.9	5.6
	Range	2.9	4.2	3.6

DIC and TA are salinity normalized. The mean (±SD) for each variable is the average of all data that falls within a given season. The range is presented as the absolute maximum minus the absolute minimum value recorded by our instrumentation for the whole season.

**Table 4:** Krustal-Wallis test results for seasonal differences in environmental parameters.

Parameter		Fall-Winter-Spring
**Temperature**	Fall (n = 141)	df = 1170
	Winter (n = 700)	X^2^ = 293.46
	Spring (n = 330)	**p<0.001**
**PAR**	Fall (n = 140)	df = 989
	Winter (n = 640)	X^2^ = 12.94
	Spring (n = 291)	**p = 0.0015**
**pH**	Fall (n = 122)	df = 853
	Winter (n = 529)	X^2^ = 139
	Spring (n = 203)	**p<0.001**
**DIC**	Fall (n = 78)	df = 220
	Winter (n = 60)	X^2^ = 1.61
	Spring (n = 84)	p = 0.4473
**TA**	Fall (n = 78)	df = 196
	Winter (n = 70)	X^2^ = 13.44
	Spring (n = 49)	**p = 0.0012**
**pCO**_**2**_	Fall (n = 78)	df = 184
	Winter (n = 58)	X^2^ = 5.44
	Spring (n = 49)	p = 0.066
**Ω**_**AR**_	Fall (n = 78)	df = 184
	Winter (n = 58)	X^2^ = 2.5
	Spring (n = 49)	p = 0.2859

p<0.05 reflects statistically significant difference across seasons based on the available data. Significant p values shown in bold.

#### Light

PAR data showed a general increase in intensity from the fall to the end of spring (Fig 2B and 2E), with a small decline during the month of October likely due to cloudiness at the beginning of the rainy season (Table 2). Seasonal PAR increased steadily during this study period (Table 3), and differences across the seasons were statistically significant (Table 4).

#### Water Depth

Water depth measurements reveal a semi-diurnal tidal cycle with a tidal range between 0.5–2.5 m during spring tides and 0.5–1.5 m during neap tides (Fig 2C). Monthly averages of water depth reveal the variability of the time of day of spring and neap tides every month (Fig 2F). Three-hour averages for pH and water depth show a small but statistically significant correlation (r = 0.07, p = 0.04). Pearson’s correlation analysis reveals there is a similarly small yet statistically significant correlation (r = 0.22, p = 0.016) between water depth and total alkalinity.

#### Salinity

**Fig 3:**
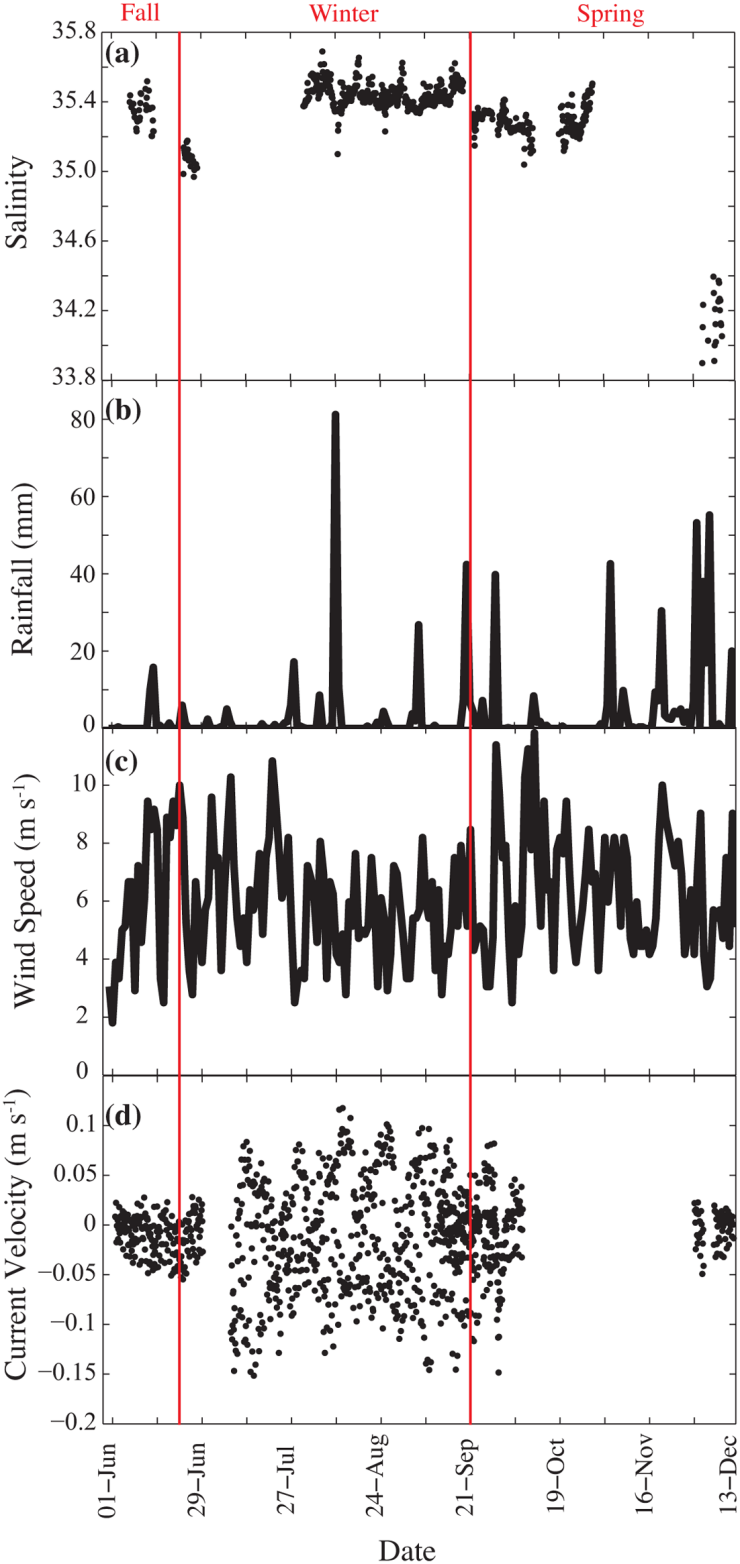
High resolution salinity, rainfall, windspeed and current velocity data. (a) Environmental data included salinity, (b) rainfall, (c) wind speed and (d) current velocity. The salinity and current velocity data were measured as part of this study while the rainfall and wind speed data are from the Australian Bureau of Meteorology (http://www.bom.gov.au/).

Salinity levels on the reef flat had a mean of 35.3 ± 0.3 psu from June through the end of October, with occasional punctuated drops in the 33.0–33.5 psu range due to rain events. Mean salinity in December was lower at 33.9 ± 0.5 psu following a sustained period of rain in November (Fig 3A and 3B).

#### Water Depth, Rainfall, Wind and Current Velocities

The spring had more regular rainy periods and higher average and maximum wind speeds (v_max_ >12 m s^-1^) in October 2010 (Fig 3B and 3C). The average rainfall was higher in the spring compared to winter, although there was one major rain event in mid-August. Southeasterly trade winds dominate this area, with westerly winds developing in the wintertime (Fig 3C). Currents on the reef flat were slowest on average in the fall and late spring with fastest along-shore velocities during these periods of around 2 cm s^-1^ to the SE and 5 cm s^-1^ to the NW, toward the harbor. In the winter, the velocities ranged from 10 cm s^-1^ to the SE to 15 cm s^-1^ to the NW (Fig 3D).

#### Nutrients

Nitrate values measured sporadically throughout the study period (n = 148) had a mean value of 0.3 ± 0.4 μmol/L while phosphate values had a mean value of 0.21 ± 0.14 μmol/L (S2 Table). The nitrate and phosphate levels are comparable to those routinely measured on oligotrophic coral reefs [50].

### Variability in Carbonate Chemistry

#### pH

**Fig 4:**
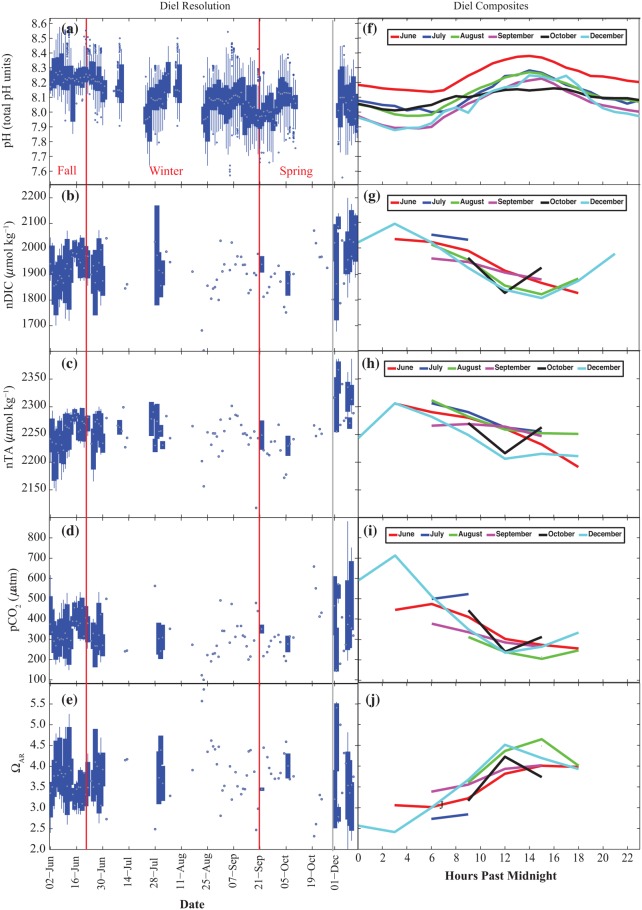
Carbonate chemistry parameters measured (pH, nDIC, nTA) and calculated (pCO_2_ and Ω_ARG_) throughout the study. (a) Hourly resolution data for pH in total pH units, (b) salinity normalized dissolved inorganic carbon (nDIC) in μmol kg^-1^, (c) salinity normalized total alkalinity (nTA) in μmol kg^-1^, (d) the partial pressure of CO_2_ (pCO_2_) in μatm and (e) the aragonite saturation state (Ω_ARG_). The edges of the box are the mid-range (25–75^th^ percentile), the lines extend to the most anomalous data points not considered outliers, and outliers are plotted individually. (f) Diel composites of the data are shown for each month for pH, (g) nDIC, (h) nTA, (i) pCO_2_ and (j) Ω_AR_.

**Fig 5:**
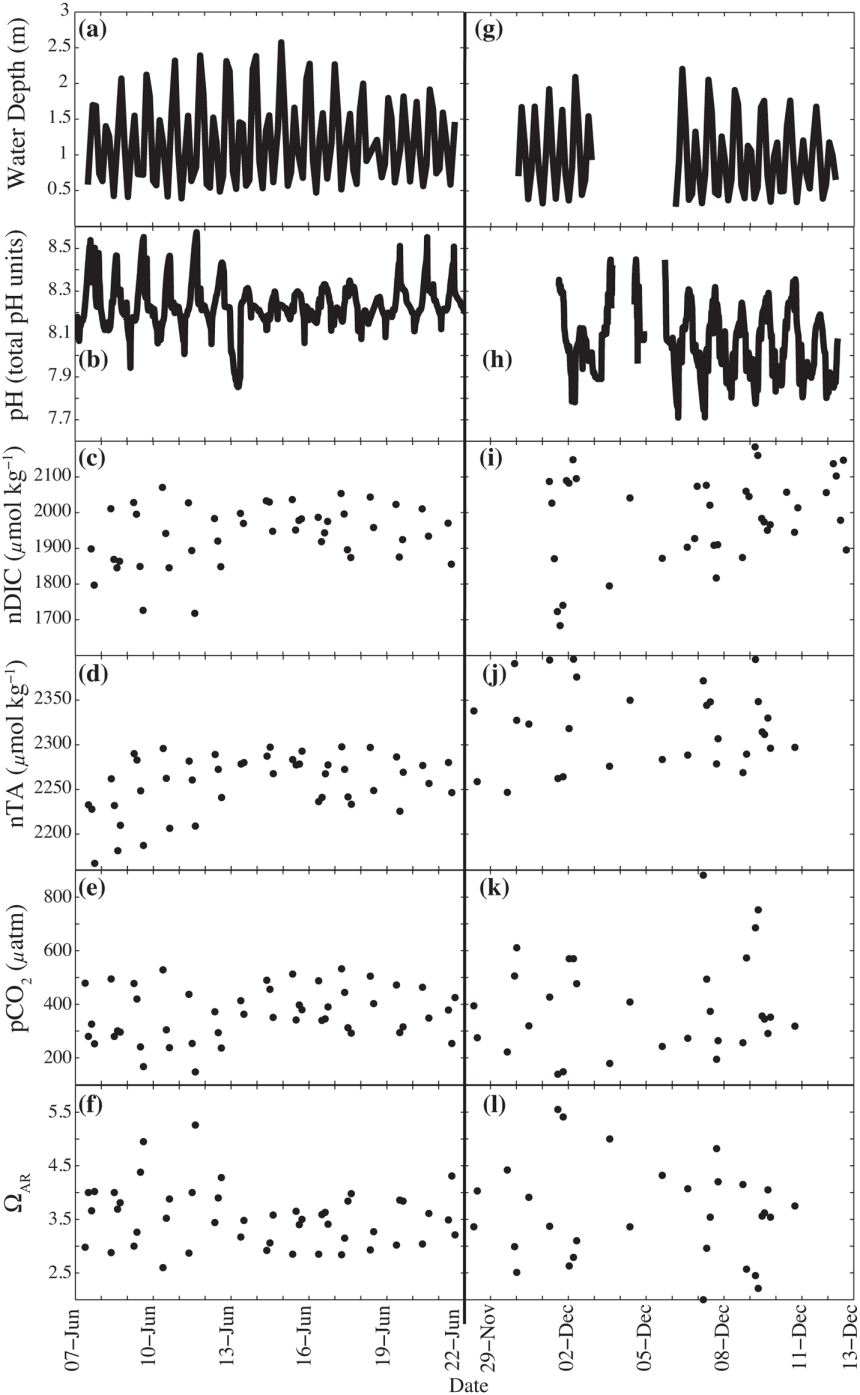
Comparison of high-resolution carbonate chemistry data during two weeks in June and December 2010. (a) The data for June 7–22, austral fall, is shown for water depth in m, (b) pH in total pH units, (c) salinity normalized dissolved inorganic carbon (nDIC) in μmol kg^-1^, (d) salinity normalized total alkalinity (nTA) in μmol kg^-1^, (e) the partial pressure of CO_**2**_ (pCO_**2**_) in μatm and (f) the aragonite saturation state (Ω_**ARG**_). (g) Data for Nov. 29—Dec 13, austral spring, for water depth, (h) pH, (i) nDIC, (j) nTA, (k) pCO_**2**_, and (i) Ω_**AR**_. During these periods, discrete samples of DIC and TA were taken at least two times per day and in some cases every 3 hours.

The highest daytime pH values occurred in the fall with a decline in pH from the fall to the winter and a rise in pH in the spring (Table 2; Fig 4A and 4F). Seasonal trends were statistically significant (Table 4), with the mean pH declining from 8.24 ± 0.1 in the fall, to 8.12 ± 0.14 in the winter to 8.04 ± 0.14 in the spring (Tables 3 and 4). The greatest diel range was observed in the winter when the average minimum and maximum pH were 7.57 and 8.61, respectively (Table 3). Periods of lowest pH occurred in the winter during the night, which is expected considering the higher CO_2_ solubility at colder water temperatures in winter and potentially greater rates of respiration at night during those times (Fig 5B and 5H).

#### nDIC

nDIC and nTA samples were taken once to twice daily, typically at noon and at the daytime low tide, throughout most of the research period because of logistical constraints, thus what we report here are observed rather than true minima and maxima (Tables 3 and 4; Fig 4B and 4G). High-frequency sampling occurred in June and during a brief period in July and in December, which allowed for the full capture of diel cycles and thus reliable comparisons between late fall and late spring conditions (Fig 5).

December had a larger diel range in nDIC values compared to June (Fig 5C and 5I; Table 2). The lowest average monthly nDIC was in mid-winter, in August, and the highest in December (Table 2). The mean seasonal nDIC increased from fall to spring with the highest seasonal maximum occurring in spring (Table 3). No statistically significant difference emerged across seasons; however, that is likely to be caused by a low number of total samples collected (Table 4).

#### nTA

nTA followed similar trends as nDIC (Fig 4C and 4H; Tables 2 and 3), with higher diel averages, minima, and maxima observed in December compared to June. The higher values and greater variability in nTA were clearly seen when comparing data from June and December (Fig 5). Mean monthly nTA values increased from late fall to spring (Table 2). The greatest and smallest diel range in nTA occurred in the spring and the fall, respectively (Table 3). The seasonal trends were statistically significant, and the seasonal means in nTA increased steadily during the study period from fall to spring (Tables 3 and 4).

#### pCO_2_

A comparison of the June to the December values shows more variability in pCO_2_ in December with higher daily values that peak at almost 900 μatm (Fig 5E and 5K). The lowest calculated pCO_2_ value was in August, as was the lowest monthly maximum (Table 2). The seasonal pCO_2_ trends were not statistically significant throughout the study period. This was also likely an artifact of the reduced discrete sampling during most of the winter and spring (Tables 3 and 4).

#### Ω_AR_

As expected, opposite trends to the pCO_2_ data were observed for Ω_AR_, with the smallest average diel means and minima observed in June (Fig 4E and 4J). A comparison of Ω_AR_ values in June vs. December show that in June there was less variation around the mean 3.6 ± 0.3, while in December there was a greater range of values with high values of 5.5 and lows of 2.0 and a mean of 3.8 ± 0.7 (Fig 5F and 5I; Table 2). Ω_AR_ seasonal means were all around 3.6 with no significant differences between fall, winter or spring (Table 4) that is also likely an artifact of the reduced discrete sampling during most of the winter and spring.

#### Thermodynamic vs. biological controls on pH and Ω_AR_

**Fig 6:**
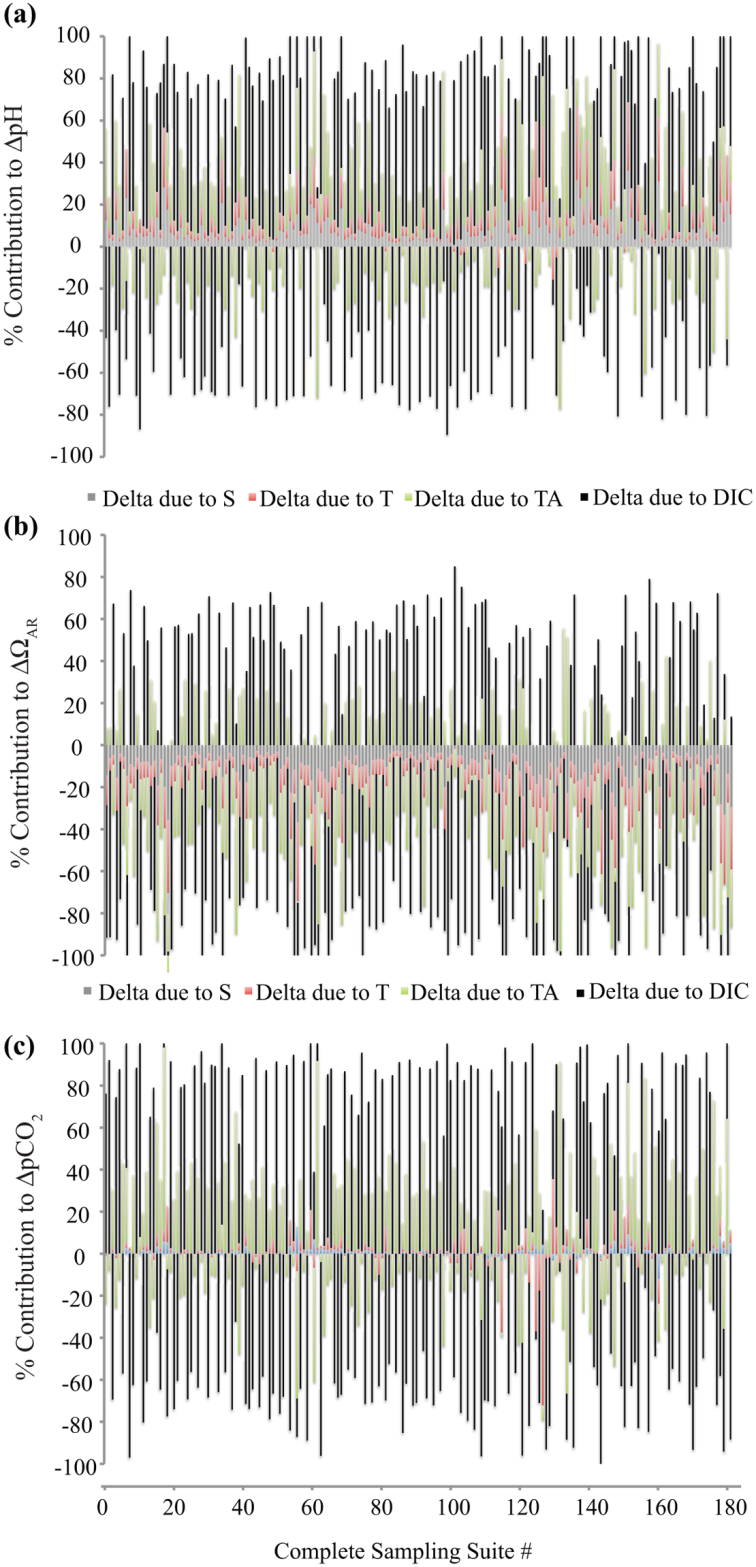
Contribution analysis to determine the role of salinity, temperature, total alkalinity and DIC in influencing different aspects of the carbonate chemistry. (a) Contributions of salinity, temperature, total alkalinity and DIC to changes in pH, (b) aragonite saturation state (Ω_**AR**_) and (c) pCO_**2**_. The complete sampling suite #s are times throughout the December 2010 study period when all of the parameters (S, T, pH, DIC, TA) were measured. The sign of percentage change (positive vs. negative) keeps track of the overall directionality of change in the pH, pCO_**2**_, or Ω_**AR**_ value.

We find that temperature and salinity have minimal contributions to pH and Ω_AR_ variability, often an order of magnitude smaller than those driven by DIC and TA changes (Fig 6A–6C). TA and DIC have predictably opposing contributions, e.g., at night increasing DIC leads to a drop in pH and Ω_AR_ and increase in pCO_2_, whereas the nighttime rise in TA counteracts the dropping pH and Ω_AR_ and rise in pCO_2_ due to reintroduction of carbonate ions. Conversely, during the day at both sites, DIC decreases due to photosynthesis and calcification leads to a rise in pH and Ω_AR_ and drop in pCO_2_, while TA consumption during calcification results in a decreased availability of carbonate ions and, hence, lowers pH and Ω_AR_ and increases pCO_2_.

#### Thermal and pH exposure metrics of intensity, duration, and severity

**Fig 7:**
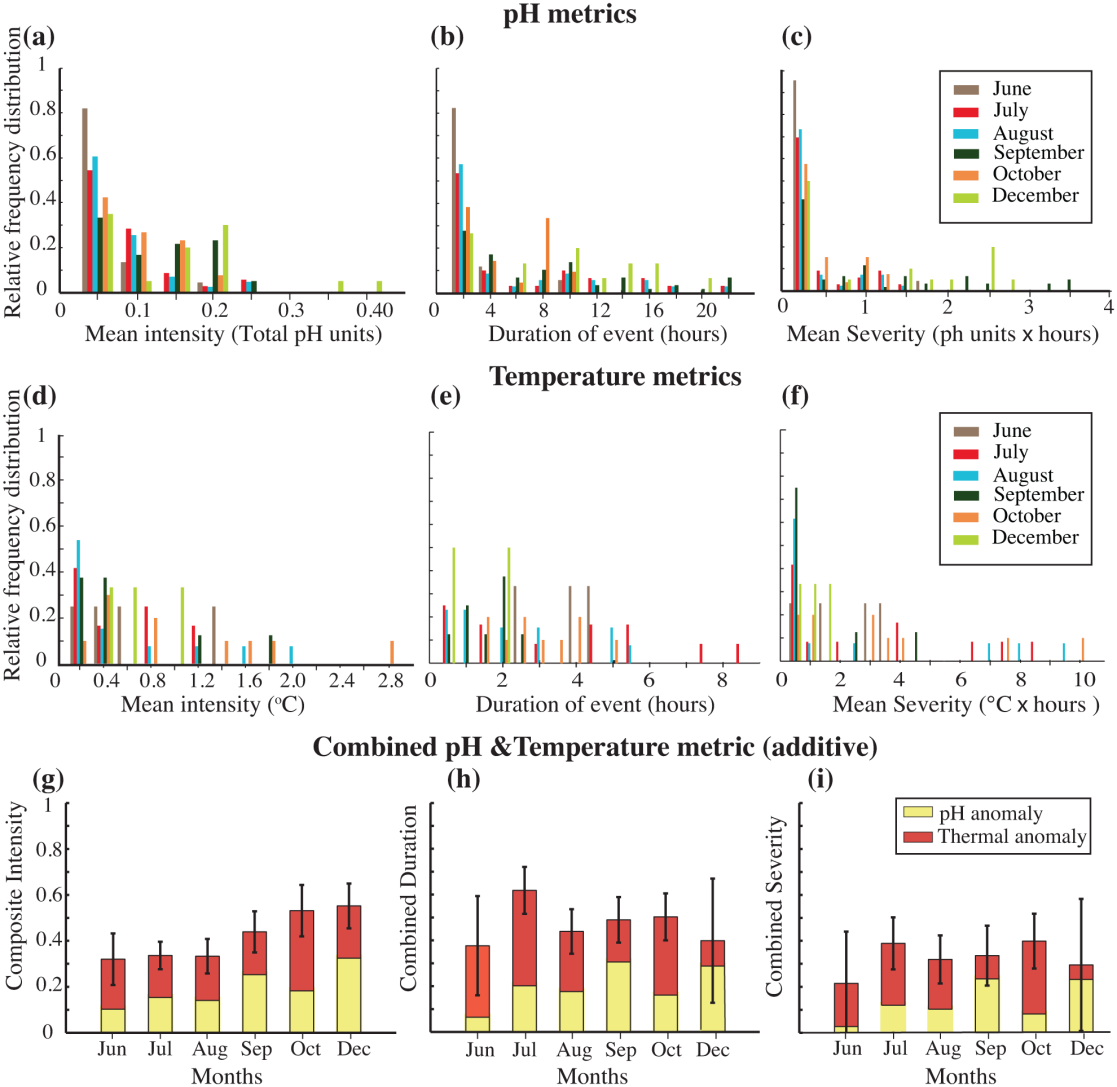
Exposure metrics for pH, temperature and an additive, combined metric for pH and temperature. (a) Frequency distribution of the mean intensity events, in total pH units, for each of the months in this study was plotted, while the duration of the events in hours was plotted in (b) and the mean severity in total ph units•hours in (c). The pH threshold (Th) was defined as 8.1 to represent the 2010 mean global open ocean pH. A similar procedure was used for the temperature data, but the mean monthly maximum temperature thresholds for each month (June-December) were determined based on temperature monitoring data available from 2008-present (Australian Institute for Marine Science, http://data.aims.gov.au/aimsrtds/station.xhtml?station=130). (d) The relative frequency distributions of monthly mean intensity of the high temperature events in °C, (e) the duration of these events in hours and (f) the mean monthly severity of these high temperature events in °C•hours. (g) The composite intensity including the mean monthly scaled pH and temperature intensity values, (h) combined duration including the mean monthly scaled pH and temperature duration values and (i) the combined severity.

The pH data suggest that there were anomalous pH conditions in September and December based on the pH 8.1 mean open ocean threshold chosen, although we had data for only part of December. There were more low-pH events in September (60 events, Table 5) than in any other month. In June we only recorded 22 of these events, of which 80% had intensities ≤ 0.05 total pH units (Fig 7A, Table 5). The highest intensities were recorded in December, with 60% of the intensities ≥ 0.15 total pH units. This was largely due to two punctuated events of high intensity values. The low pH events in June were shortest, with 80% lasting 2 hours or less, while 40% and 60% of the low pH events in September and December respectively, lasted 10 hours or longer (Fig 7B). As a function of duration and intensity, September and December were the two months with the highest low-pH event severity during the study, while June had the lowest severity (Fig 7C).

**Table 5:** Mean and standard error of intensity (I), duration (D), and severity (S) of pH, temperature and combined events.

	June	July	August	September	October	December
pH, mean intensity	0.039 ± 0.01	0.06 ± 0.01	0.05 ± 0.01	0.09 ± 0.09	0.07 ± 0.01	0.1210 ± 0.0235
pH, number of events	22	35	43	60	26	20
pH, measurements below threshold	131	656	739	1734	431	535
pH, mean duration (hours)	1.49 ± 0.4	4.69 ± 0.95	4.1 ± 0.81	7.1 ± 0.8	3.74 ± 0.67	6.69 ± 1.42
pH, mean severity	0.11 ± 0.07	0.5 ± 0.17	0.43 ± 0.14	0.98 ± 0.14	0.34 ± 0.08	0.97 ± 0.23
T, mean intensity	0.5725 ± 0.2723	0.4816 ± 0.1082	0.5071 ± 0.1723	0.4911 ± 0.2109	0.9192 ± 0.2624	0.5992 ± 0.1960
T, number of events	4	12	13	8	10	3
T, number of measurements above threshold	45	180	123	53	123	12
T, mean duration	2.81 ± 0.95	3.75 ± 0.83	2.37 ± 0.51	1.66 ± 0.23	3.08 ± 0.38	1 ± 0.52
T, mean severity	1.85 ± 0.72	2.66 ± 0.87	2.14 ± 0.94	1.0 ± 0.53	3.13 ± 1	0.62 ± 0.29
Combined T & pH intensity	0.32 ± 0.11	0.34 ± 0.06	0.33 ± 0.08	0.44 ± 0.09	0.53 ± 0.11	0.55 ± 0.1
Combined pH & T duration	0.38 ± 0.22	0.62 ± 0.1	0.44 ± 0.1	0.49 ± 0.1	0.5 ± 0.1	0.4 ± 0.27
Combined pH & T severity	0.21 ± 0.23	0.39 ± 0.11	0.32 ± 0.1	0.34 ± 0.13	0.4 ± 0.12	0.29 ± 0.29

The pH threshold used was 8.1 based on the 2010 mean global open ocean pH, while the Mean Monthly Maxima (MMM) temperature thresholds used for June, July, August, September, October, and December were 23.81°C, 23.12°C, 23.93°C, 25.32°C, 25.98°C, and 29.0°C, respectively. I, D, and S metrics are presented for temperature and pH individually, as well as in a composite metric that incorporates both stressors. pH and temperature metrics for intensity, duration, and severity are shown as raw, unscaled averages for each month, based on the I_pH_; however, the combined metrics are shown as scaled averages (refer to Methods section for more details).

The temperature conditions were arguably most anomalous in July, August and October (Table 5, Fig 7). The temperature data reveal that October had the greatest number of intense high-temperature events with approximately 40% of the exposure events with a mean intensity ≥1.2°C and 60% with a mean intensity ≥0.8°C. In December, 60% of the high-temperature events had a mean intensity between 0.8–1.2°C, while in October 40% were between 0.8–2.0°C (Fig 7D). The longest duration events occurred in the austral winter, June—August, with 60% of the temperature events lasting 4–8 hours in July and 80% in June lasting 3–4 hours (Fig 7E). In spring, high-temperature events were generally more intense but lasted only two hours or less in September and December. The most severe high-temperature events were in October, August and July, respectively (Fig 7F).

The combined environmental intensity, including values for both pH and temperature, increased from June to December with the highest combined intensity events in December, followed by October and September. The late fall (June) and winter months (July, August) had the lowest combined intensities (Fig 7G, Table 5). There was no seasonal pattern in the combined duration and severity of events (Fig 7H and 7I). The combined duration of events was longest in July, followed by October, largely driven by long-lived thermal exposure events. June exhibited the shortest combined duration of events. Even without the combined exposure metric, it was clear that the periods of highest intensity and severity of pH exposure events (September and December) corresponded to times of least intense and least severe thermal exposure events, and vice versa.

## Discussion

We contribute two rigorous analytical approaches with this paper: 1) the assessment of relative contribution of thermodynamic and biological drivers to variability in pH, aragonite saturation state, and pCO_2_, which provides further evidence that not only do reefs modulate their own reef water carbon chemistry, but they also effectively play a role in determining the exposure, and 2) the development of metrics for exposure to pH and temperature, which are two factors known to play an important role in physiological and ecological processes.

### Relative Contribution of Thermodynamic and Biological Drivers

The biologically driven DIC and TA variability is a stronger driver of pH, Ω_AR_, and pCO_2_ than salinity and temperature, at this location (Fig 6). Since DIC and TA are driven primarily by reef metabolism processes, their influence on the variability of pH, Ω_AR_, and pCO_2_ suggests that changes in NEC and NEP would have a significant biogeochemical feedback on the diel variability and average diel values for pH and Ω_AR_ on the reef in the future. Salinity-based reconstructions of carbonate system parameters, which are routinely used to calculate alkalinity in the open ocean (e.g., [51]), would not produce accurate representations of the environmental variability in aragonite saturation state or other carbonate system parameters in coral reefs.

### Environmental Exposure Metrics for pH and Thermal Stress

Long-term observations such as these presented here from the Heron reef flat, as well as others, for example for various reef zones from Palmyra Atoll [52], raise the question what environmental metrics, for pH, temperature, and other variables, are most relevant to the physiological and ecological processes on reefs that may be affected by continued OA. Price *et al*. (2012) [52] provide estimates for duration of pH conditions above a certain threshold by using the metric of number of hours when pH stays above a site-specific climatological seasonal low (CSL) value. The authors find that net calcification rate and percent cover usually increase as the number of hours of pH exposure above the CSL increase [52]. Our approach presented here used exposure in a different way, investigating intensity and duration of exceedence of a pH minimum threshold as well as temperature maximum threshold, thus adding one more way to examine pH variability on reefs as it may relate to vital ecological processes in these settings.

We used metrics of intensity, duration and severity developed for coral bleaching temperature data [53] and adapted it for carbonate chemistry data [49] for both pH and temperature stress alone and for a combined metric to try to better determine which months were likely more physiologically stressful during the 6-month study period. This analysis suggests that highly intense events of high severity generally do not occur simultaneously for high temperature and low pH exposure, especially at the end of fall (June) and end of spring (December) at Heron Island. This could have both positive and negative implications for the vulnerability of reef flats to climate changes as asynchronous impacts may allow reef organisms time to recover from low pH or high temperature exposure and possibly minimize synergistic impacts. However, the potential negative impacts could be that with asynchronous exposure there is less time during the year when the reef organisms are not exposed to potential stressful environmental conditions increasing the possibility that a sensitive life stage event such as reproduction or settlement occur under challenging environmental conditions.

Environmental exposure analysis can provide context with respect to how different reef habitats are conditioned by their environment. While a combined pH and thermal exposure metric has not been developed before, we view this study as an important effort to understand coupled environmental pressures associated with global change, and we recognize the limitations of the approach used here. The results are sensitive to the threshold values chosen, as was seen in [49], and we used a pH threshold of 8.1, based on the 2010 mean global open ocean pH [31], as a conservative first approach. In the future, a pH threshold based on long-term pH data for the site would be more appropriate for determining internal thresholds similarly to the way temperature thresholds were determined in this study. It will also be important to integrate additional environmental parameters such as PAR, additional carbonate chemistry parameters and nutrients into the combined exposure metrics. Furthermore, the combined metric assumes that the interactive effects between temperature and pH stress are additive but it is also possible that they could be synergistic with variable contributions, antagonistic or even have no interaction [54–56]. It is clear that more studies will be needed to determine the appropriate method to determine a combined metric for pH and temperature. Future studies, likely site-by-site and species-by-species, will further elucidate the distinctly different weight of pH and thermal stress contributions to organisms’ sensitivity and resistance to global change. Such future work will enable improved estimates for a combined exposure metric that will be useful for predicting and managing times with high environmental exposure severity.

The life-stage, energy reserve status and acclimation strategies of reef flat organisms will largely determine the respective physiological and ecological importance of the temperature and pH exposure events. For example, key life-stage events for reef flat corals include timing of mass spawning events, larval recruitment and settlement, as well as allocation of energy to reproductive output. Studies have shown that coral species experience energy trade-offs between different physiological processes (e.g., calcification, reproduction, etc.) when exposed to environmental stress [57, 58]. Coral spawning research at the Heron Island Research Station suggests that corals at this location typically spawn between November and January [59, 60]. Using coral and crustose coralline algae species found on Heron reef, research has shown that coral recruitment can be significantly hampered under future predicted OA conditions (pH 7.6–7.8) [60]. If lowered pH conditions on the Heron reef occur during a critical part of the coral reproductive cycle, it is possible that corals could sacrifice calcification rate in order to maintain reproductive success. This could potentially lead to corals directing energy away from reproduction in order to maintain calcification under OA conditions or potentially shifting the timing of their spawning events. Our data show that during November-December the alkalinity is higher than the rest of the sampling period, which may be indicative of lower calcification rates. Lower calcification rates may be due to coral spawning events during this period, but could also be due to high cloud cover associated with decreasing light-enhanced calcification rates. Combined low pH, high temperature events will likely become more frequent in a high-CO_2_ future and as both the severity and duration of these stressful conditions increase, their potential impact may grow. However, there is emerging evidence that some coral reef environments (e.g., reef flats in Ofu, American Samoa) have been able to survive under conditions previously classified as severe exposure [61]. Emerging research shows that biogeochemical feedback from declining calcification and increased photosynthesis on reefs may actually counteract expected pH declines in the future to some extent, yet at significant cost to coral reef health and ecosystem function [47].

### Comparison of Seasonal Variability in Carbon Biogeochemistry

To provide more context for the seasonality we observe in this study, we compare our results from Heron Island in 2010 with studies from Media Luna reef, Puerto Rico in 2007–2008 [62]; Lady Elliott Reef flat, Great Barrier Reef from 2009–2010 [22]; and the Lower, Middle, and Upper Keys of the Florida Reef Tract from 2009–2010 [63] (Table 6). Seasonal differences in carbon system parameters are assumed to be driven by the combination of the biogeochemical signature of incoming offshore waters, seasonality in key reef biological processes that influence the local carbonate chemistry including calcification/dissolution and photosynthesis/respiration, and changes in reef hydrodynamics and residence time [64]. Comparing the results from the 4 reef studies confirms that Lady Elliott Island (LEI), a similar reef flat site on the GBR, had similar seasonal ranges in environmental variables to the Heron Island study as expected (Table 6). The temperature ranges are similar for these two GBR locations, but the absolute temperatures are generally lower at Heron Island than LEI, except in the winter, when the temperatures are quite similar at both sites. The salinity ranges for Heron Island and LEI are also similar to each other across the seasons: greater in the winter and spring, smaller in the summer and fall. The pH ranges for Heron Island and LEI are very similar, and the Heron Island pH seasonal average is greater than the Media Luna reef average during every season, but winter was characterized by the highest diel pH maxima in both GBR sites as well as at Media Luna.

**Table 6:** Comparison between studies showing seasonal patterns in temperature, salinity, and carbon system parameters.

Measured Variable	Season	Gray et al. 2012 (Media Luna reef, Puerto Rico); data from 2007–2008	Manzello et al. 2012 (Florida Reef Tract); data from 2009–2010	Shaw et al. 2012 (Lady Elliott reef, GBR); data from 2009–2010	This study (Heron Island reef, GBR); data from 2010
**Temp. (°C)**	Spring	N/A	N/A	21.9–25.4 (3.5) §	20.4–29.1 (23.4 ± 1.5)** (8.7) §
	Summer	29.3 ± 0.2	N/A	25.0–31.2 (6.2) §	N/A
	Fall	29.1 ± 0.5	N/A	23.1–28.7 (5.6) §	18.5–24.1 (22.0 ± 1.1)** (5.6) §
	Winter	26.3 ± 0.4	N/A	17.6–25.9 (8.3) §	17.9–26.0 (21.9 ± 1.1)** (8.1) §
**Salinity**	Spring	N/A	36.7–37 (0.3) §	32.3–35.5 (3.2) §	32.1–35.5 (35.1 ± 0.5)** (3.4) §
	Summer	35.8 ± 0.22	36.6–37.3 (0.7) §	34.9–35.8 (0.9) §	N/A
	Fall	33.8 ± 0.5	35.9–36.2 (0.3) §	34.8–35.6 (0.8) §	33.3–35.5 (35.1 ± 0.6)** (2.2) §
	Winter	35.2 ± 0.35	35.8–36.1 (0.3) §	34.3–35.8 (1.5) §	32.9–35.7 (35.4 ± 0.2)** (2.8) §
**pH (total scale)**	Spring	N/A	N/A	7.59–8.29 (0.7) §	7.66–8.45 (8.04 ± 0.14)** (0.79)§
	Summer	8.01 ± 0.02	N/A	7.60–8.34 (0.74) §	N/A
	Fall	8.00 ± 0.03	N/A	7.69–8.49 (0.8) §	7.85–8.58 (8.24 ± 0.1)** (0.7) §
	Winter	8.09 ± 0.02	N/A	7.74–8.56 (0.82) §	7.57–8.61 (8.12 ± 0.14)** (0.94) §
**nTA (μmol kg** ^**-1**^ **)**	Spring	N/A	2100.8–2195.1 (94.3) §	2169–2374 (205) §	2118–2387 (2273 ± 56)** (269) §
	Summer	2315 ± 6*	2059.2–2207.5 (148.3) §	2122–2538 (416) §	N/A
	Fall	2223 ± 30*	2277.1–2316.1 (39) §	1922–2429 (507) §	2148–2298 (2242 ± 39)** (150) §
	Winter	2295 ± 39*	2384.4–2410.1 (25.7) §	2012–2397 (385) §	2156–2309 (2259 ± 31)** (153) §
**nDIC (μmol kg** ^**-1**^ **)**	Spring	N/A	1743.2–1854.1 (110.9) §	1783–2234 (451) §	1677–2199 (1952 ± 118)** (522) §
	Summer	1996 ± 10*	1720.9–1840.8 (119.9) §	1636–2394 (758) §	N/A
	Fall	1921 ± 21*	1987.7–2023.8 (36.1) §	1450–2264 (814) §	1701–2071 (1913 ± 94)** (370) §
	Winter	1974 ± 32*	2042.5–2054.0 (11.6) §	1412–2174 (762) §	1603–2073 (1931 ± 92)** (470) §
**pCO**_**2**_ **(μatm)**	Spring	N/A	257–316 (59) §	186–1271 (1085) §	139–882 (374 ± 163)** (743) §
	Summer	460 ± 33	338–379 (41) §	150–1325 (1175) §	N/A
	Fall	437 ± 44	395–452 (57) §	89–996 (907) §	148–528 (344 ± 96) ** (380) §
	Winter	356 ± 43	299–330 (31) §	70–892 (822) §	100–867 (326 ± 126) ** (767) §
**Ω**_**AR**_	Spring	N/A	4.07–4.69 (0.62) §	1.13–4.84 (3.71) §	2.0–5.6 (3.6 ± 0.8) ** (3.6) §
	Summer	3.94 ± 0.24	3.90–4.32 (0.42) §	1.45–6.08 (4.63) §	N/A
	Fall	3.42 ± 0.26	3.42–3.47 (0.05) §	1.59–6.46 (4.87) §	2.4–5.3 (3.6 ± 0.6) ** (2.9) §
	Winter	3.94 ± 0.25	3.91–4.08 (0.17) §	1.62–6.01 (4.39) §	1.7–5.9 (3.7 ± 0.7) ** (4.2) §

Shaw et al. 2012, Manzello et al. 2012, and this study show ranges observed across multiple seasons. The ranges for Manzello et al. 2012 are for in-shore sites in the Upper, Middle, and Lower Keys. Gray et al. 2012 show means;

* Gray et al. 2012 show calculated nDIC and nTA, whereas in all other studies, the nDIC and nTA are directly measured.

** represent the mean and standard deviation (mean ± SD) where available;

^§^ represent the seasonal range where available.

Seasonal ranges in TA for the Florida Reef Tract (FRT) were an order of magnitude smaller than those for the GBR sites regardless of the season and could be due to a number of factors including a shorter seawater residence time, less coral coverage leading to less calcification, and increased dissolution along the FRT or a combination of these and other factors. The alkalinity ranges for LEI, however, were much greater than those for Heron Island, suggesting greater calcification/dissolution rates and/or greater residence time of LEI waters. The pCO_2_ maxima and the seasonal ranges are much higher for the GBR sites compared to the FRT, which correspond to the smaller ranges in the DIC and TA, suggesting that this may be an effect of the different reef characteristics and the chemical environment of the surrounding seawater feeding the reefs. The lowest seasonal averages for pCO_2_ in all sites were observed in the winter (Table 6). Lastly, the Ω_AR_ ranges were greater across all seasons for the GBR sites compared to the FRT and the Puerto Rico sites, with LEI having the greatest ranges and the lowest minima. While Ω_AR_ values are greater than 3.3 all the time for the FRT and most of the time for the Puerto Rico reef at Media Luna, the Ω_AR_ values for the reef flats of the GBR reached minima close to an aragonite saturation state value of 1 quite frequently at night during every season, most markedly during the fall for LEI and the winter for Heron Island. These comparisons demonstrate that seawater chemical properties differ by reef location, reef type, residence time, ecological community composition, as well as calcification/dissolution and photosynthesis/respiration rates for the reef community.

### Future Research Directions

This study found similar diel and seasonal trends as those observed on other reef flats and contributes to a number of such field studies [22, 23, 33, 62, 63, 65]. This 6-month high-resolution data set from the Heron Island reef flat reveals that there were large daily, weekly, monthly and seasonal variations in the environmental conditions measured.

Coral reefs are among the ecosystems most sensitive to OA impacts [66], yet the diel variability in carbonate chemistry on shallow reef zones often exceeds levels predicted for 2100 in the open ocean [12, 31], and coral calcification rates have already been observed to be sensitive to carbonate chemistry in much smaller pH ranges than those expected for the end of the century [7, 67]. High-frequency carbon system field studies are needed over longer time scales across different reef zones to help improve projections for future reef conditions in warmer and acidifying seas and help elucidate reef zone vulnerability. The urgent need for targeted reef biogeochemical monitoring efforts have been recognized as an ecosystem-based management priority [68] and will require a combination of CO_2_ buoy networks, autonomous instrumentation platforms that monitor at least two carbonate chemistry parameters autonomously, typically pH and pCO_2_, as well as regular discrete sampling for DIC and TA. Recent studies have also demonstrated the importance of combining high-resolution biogeochemical measurements with hydrodynamic characterization in order to best quantify the drivers of carbonate chemistry variability and the modulators of biological activity, reef metabolism and ecological community composition [25, 33]. Future studies should focus on temporal and spatial variability of carbonate chemistry and physiological responses of corals to document the heterogeneity of reef environments and to provide a better understanding of controls on carbon cycling, reef calcification, and aspects of reef resilience [69, 70].

Furthermore, high-resolution carbonate data is necessary to ground-truth, force and evaluate regional biogeochemical models, which could be used as an additional tool to analyze the drivers and magnitude of carbonate chemistry variability and to project how ocean acidification and climate change may affect the biogeochemical conditions in the future [48]. With more, long-term high-resolution carbonate chemistry data it will become possible to incorporate it into a reef health model such as NOAA’s Coral Reef Watch [71] so that a combined thermal and pH stress predictive model could be developed.

Ocean acidification, warming and other multi-stressor experimental studies should attempt to incorporate the natural diel and seasonal variability in their controls and treatments as it will likely have a major impact on the results. A recent study on Heron Island [17] incorporated the natural reef variability by reproducing values of temperature and pCO_2_ measured from a reef buoy as their control treatments, and performed all pre-industrial and future treatments as an offset from these measured natural reef conditions. Additionally, recent field, *in situ* experimental studies have started incorporating natural variability in key ecosystem variables [41]. Such work indicates natural environmental variability needs to be closely replicated in experimental design since tracking environmental variability will likely impact coral reef physiological and ecological responses, will ensure more biologically-relevant results, and will be critical to understanding the impacts of local and global stressors in a high CO_2_ future.

## Conclusion

Quantifying the temporal dynamics in temperature, pH, and other variables on diel, monthly, and seasonal scales can indicate times where the coral reef community experiences potentially anomalous events such as low pH or high temperature conditions. Future research pairing such exposure information involving intensity, duration, and severity of events with information on physiological processes on reefs and their ecological community composition can provide crucial insights into how environmental dynamics confer or hinder resilience to a coral reef community in future warming and acidifying seas. We provide a method to create exposure metrics individually for pH and temperature as well as a combined additive exposure metric. Future work should be focused on improving environmental exposure metrics and demonstrate site-specific approaches to quantifying variable contributions from different variables, most importantly, the relationship between global stressors, e.g. pH and temperature, and local stressors, e.g. nutrient loading and sedimentation. While temperature is an exogenous environmental variable, a physical variable that coral reef organisms are passively subjected to, pH is a variable that has not only physical drivers but is also modulated by the coral reef ecosystem calcification processes as well.

In essence, to a large extent reefs modulate their own exposure to pH, especially in back-reef zones that are sometimes isolated from open ocean water. Reefs are both experiencing pH diel cycles, but they are also contributing to the actual range of the diel cycle. Studying the dynamics of diel cycles for long periods of time on coral reefs can help us better understand how different reefs can modulate their carbon system environment in different ways and how these modulations may interact to produce variable rates of ocean acidification on different reefs.

## Supporting Information

S1 Table Sea-surface temperature (SST) monitoring data available from 2008-present for 11 sensors at various locations on the Heron Island lagoon and reef flat (Australian Institute for Marine Science, http://data.aims.gov.au/aimsrtds/station.xhtml?station=130). From each of the 11 sensors, the maximum temperature was extracted for each month and then averaged across the 11 sensors to get a single maximum sea surface temperature (SST) for Heron Island reef representative of each month of each year. Then, a Mean Monthly Maximum (MMM) time series of 12 values was calculated based on the temperature data from 2008–2013.
(PDF)

S2 Table Nutrient data measured from discrete seawater samples throughout the study period. (PDF)
