# Supplementary material for: Six Month In Situ High-Resolution Carbonate Chemistry and Temperature Study on a Coral Reef Flat Reveals Asynchronous pH and Temperature Anomalies
Source: PLoS One. 2015 Jun 3;10(6):e0127648. doi: 10.1371/journal.pone.0127648 (PMC4454517; doi:10.1371/journal.pone.0127648)
Supplement: S1 Table — From each of the 11 sensors, the maximum temperature was extracted for each month and then averaged across the 11 sensors to get a single maximum sea surface temperature (SST) for Heron Island reef representative of each month of each year. Then, a Mean Monthly Maximum (MMM) time series of 12 values was calculated based on the temperature data from 2008–2013. (PDF) [file pone.0127648.s001.pdf]

S1 Table

Sea-surface temperature (SST) monitoring data available from 2008-present for 11 sensors at various locations on the Heron Island lagoon and reef flat (Australian Institute for Marine Science, http://data.aims.gov.au/aimsrtds/station.xhtml?station=130). From each of the 11 sensors, the maximum temperature was extracted for each month and then averaged across the 11 sensors to get a single maximum sea surface temperature (SST) for Heron Island reef representative of each month of each year. Then, a Mean Monthly Maximum (MMM) time series of 12 values was calculated based on the temperature data from 2008-2013.

Month	Temperature (°C)	Years SST data available
January	29.41	2009-2013
February	29.91	2009-2013
March	29.20	2009-2013
April	28.19	2009-2013
May	25.65	2009-2013
June	23.81	2009-2012
July	23.12	2009-2013
August	23.93	2009-2013
September	25.32	2009-2013
October	25.98	2009-2013
November	27.64	2009-2012
December	29.00	2008-2012
