# Supplementary material for: Six Month In Situ High-Resolution Carbonate Chemistry and Temperature Study on a Coral Reef Flat Reveals Asynchronous pH and Temperature Anomalies
Source: PLoS One. 2015 Jun 3;10(6):e0127648. doi: 10.1371/journal.pone.0127648 (PMC4454517; doi:10.1371/journal.pone.0127648)
Supplement: S2 Table — (PDF) [file pone.0127648.s002.pdf]

S2 Table

Nutrient data measured from discrete seawater samples throughout the study period.

Julian Day Time	Nitrate (µmol L^{-1})	Phosphate (µmol L^{-1})
149.64583	1.2	0.33
150.65972	1.2	0.18
151.40694	0.7	0.20
151.68611	0.2	0.11
151.76528	0.6	0.24
152.25	0.5	0.18
152.38056	0.7	0.23
152.51736	0.8	0.23
152.63472	0.1	0.15
152.72083	0.3	0.13
152.90694	0.0	0.67
153.37361	0.5	0.18
153.49931	0.5	0.22
153.62222	0.5	0.17
153.70556	0.3	0.14
154.24514	0.3	0.14
154.38056	0.2	0.15
154.50556	0.4	0.14
154.62361	0.4	0.15
154.7375	0.1	0.18
155.38056	0.1	0.16
155.50556	0.5	0.16
155.62569	0.8	0.16
155.75139	0.5	0.56
156.38194	0.0	0.19
156.5	0.5	0.15
156.63194	0.6	0.15
157.37569	0.1	0.11
157.5	0.2	0.13
157.63194	0.4	0.14
158.37917	0.1	0.26
158.49931	0.1	0.11
158.62361	0.2	0.13
159.37639	0.1	0.16
159.50208	0.1	0.11
159.62569	0.4	0.13
159.7375	0.3	0.12
160.37917	0.3	0.13
160.50069	0.3	0.13
160.62639	0.9	0.15
160.72639	0.6	0.14
161.25903	0.2	0.12
161.37361	0.1	0.11
161.50903	0.2	0.13
161.62292	0.3	0.10
162.37708	2.5	0.66
162.50417	0.1	0.09
162.625	0.3	0.10
163.37569	0.3	0.12
163.5	0.2	0.15
163.62361	0.3	0.11
164.375	0.1	0.11
164.50347	0.1	0.11
164.625	0.1	0.07
165.375	0.2	0.14
165.49444	0.1	0.11
166.37569	0.2	0.14
166.49722	0.2	0.09
166.61875	0.2	0.22
167.37222	0.1	0.09
167.50417	0.1	0.10
167.62847	0.0	0.09
167.73194	0.1	0.08
168.37431	0.3	0.16
168.49861	0.3	0.15
168.625	0.1	0.11
168.73403	0.2	0.11
169.25694	0.2	0.12
169.37431	0.4	0.13
169.50347	0.5	0.09
169.62847	0.6	0.12
170.36458	0.1	0.10
170.49375	0.4	0.13
171.37153	0.2	0.15
171.49792	0.2	0.17
171.625	0.2	0.12
172.375	0.0	0.11
172.62083	0.1	0.10
173.37431	0.1	0.13
173.62847	0.2	0.15
178.53611	0.2	0.24
178.64583	0.6	0.20

179.38542	0.0	0.13
179.51944	0.2	0.26
179.64236	0.2	0.23
180.38889	0.2	0.14
180.53194	0.1	0.19
180.66736	0.1	0.15
181.52917	0.5	0.15
181.70903	0.4	0.15
190.00417	0.4	0.35
190.53611	0.1	0.12
191.57708	0.1	0.18
192.44653	0.0	0.19
193.67569	0.3	0.26
194.66111	0.2	0.28
207.37292	0.0	0.37
207.61042	0.0	0.18
208.39306	0.0	0.18
208.65417	0.1	0.12
209.67222	0.3	0.13
210.38889	0.4	0.16
210.67431	0.2	0.12
211.46319	0.3	0.15
211.69792	0.2	0.13
212.48681	0.2	0.18
213.51736	0.2	0.20
213.75625	0.2	0.15
215.35903	0.0	0.13
217.38819	0.2	0.33
229.38889	0.0	1.07
234.61875	0.1	0.35
235.65208	0.2	0.34
237.57986	0.1	0.61
239.63264	0.2	0.29
240.69167	0.1	0.46
241.69444	0.0	0.20
242.64236	0.0	0.27

243.71667	0.0	0.36
244.35625	0.0	0.27
245.44375	0.3	0.31
249.5	0.1	0.26
250.5	0.2	0.31
252.51319	0.1	0.25
270.26597	0.1	0.24
279.59236	0.0	0.08
280.56944	0.1	0.16
280.71667	0.3	0.18
281.55208	0.1	0.14
283.7	0.1	0.13
294.46528	0.3	0.29
297.46181	0.1	0.16
298.46319	0.2	0.16
305.45694	0.1	0.18
310.46319	0.1	0.20
312.47222	0.0	0.19
315.47569	0.0	0.18
317.47569	0.0	0.51
332.35417	1.6	0.42
333.35903	1.9	0.41
333.49306	0.4	0.24
334.38194	0.6	0.80
334.93056	0.2	0.17
335.47917	0.8	0.20
336.28125	0.1	0.14
342.46667	0.2	0.37
343.72917	21.9	3.61
344.21042	0.5	0.21
344.31736	1.6	0.30
344.46667	0.1	0.16
344.69167	1.4	0.30
345.42361	1.1	0.70
